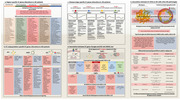# Heterogeneity of brain endothelial cell transcriptome in Alzheimer’s disease brain tissue

**DOI:** 10.1002/alz.092105

**Published:** 2025-01-03

**Authors:** Qian Yue, Pui Man Maggie Hoi

**Affiliations:** ^1^ Department of Pharmaceutical Sciences, Faculty of Health Sciences, University of Macau, Macau, Macau China; ^2^ State Key Laboratory of Quality Research in Chinese Medicine, Institute of Chinese Medical Sciences, University of Macau, Macau, Macau China; ^3^ The fifth Affiliated Hospital of Jinan University (Heyuan Shenhe people’s Hospital), Heyuan, Guangdong China

## Abstract

Brain endothelial cell (BEC) dysfunction in Alzheimer’s disease (AD) have attracted much more attention in recent years. A series of multi‐omics study is carried out to indicate the specific endothelial state during AD progression. We have summarized the most recent AD multi‐omics study on AD BECs. This figure shows multiple characterization of transcriptomic information. BEC transcriptomic alteration shows region‐specific and subpopulation‐specific manner, in which different groups of differentially expressed genes (DEGs) are detected in each category, as well as related signaling pathways and biological processes. DEGs and related biological processes also shows changing trends as the disease progresses from no neuritic plaques (Braak0‐II) to severe neuritic plaques (BraakVI), which indicates that the pathologic process in AD is dynamic. Then we show the potential downstream and upstream factors related to BEC DEGs, including BEC receptors, transcription factors and AD GWAS loci. Finally, we show the DEGs as well as related biological processes in BECs near the Aβ plaques, or in cerebral amyloid angiopathy (CAA). An overlap between microglia risk GWAS and BMECs DEG is noticed and shown. In addition, BEC DEGs also indicate the activation of BEC receptors that may be related to several AD ligands produced by other brain cells like oligodendrocytes, astrocytes, pericytes, and neurons, showing the close relationship between BEC pathology and other cell pathology. Taken together, BEC transcriptomic information strongly indicated the close relationship between BEC DEGs and AD pathogenesis and is valuble for the exploration of therapeutic strategies for AD by targeting BEC dysfunction.